# Splenic infarction as a complication of covid-19 in a patient without respiratory symptoms: A case report and literature review

**DOI:** 10.1016/j.idcr.2021.e01062

**Published:** 2021-03-20

**Authors:** Gustavo Rodrigues Alves Castro, Iwan Augusto Collaço, Caroline L. Balcewicz Dal Bosco, Gustavo Gusso Corrêa, Giovana Balcewicz Dal Bosco, Giovana Luiza Corrêa

**Affiliations:** Department of General Surgery, Hospital do Trabalhador, Av. Rep. Argentina, 4406 - Novo Mundo, Curitiba, Paraná, 81050-000, Brazil

**Keywords:** Splenic infarction, COVID-19, SARS-CoV-2, Disseminated intravascular coagulation

## Abstract

**Introduction:**

Multiple studies suggest that SARS-CoV-2 infection is associated with a pro-thrombotic state and thrombotic events have been recorded in several organs and systems. We report a patient with no respiratory symptoms, presented with abdominal pain and an extensive splenic infarction after COVID-19.

**Case report:**

A 67 year-old man was admitted to the emergency department with a moderate, dull, left-sided abdominal pain. The patient denied respiratory symptoms but referred contact with family members positive for COVID-19. He tested positive for COVID-19 and had increased D-dimer levels. Imaging studies revealed splenic infarcts and ground-glass opacities in bilateral pulmonary bases. He was treated with full-dose anticoagulation and was discharged home on oral Rivaroxaban.

**Discussion:**

Although rare in the literature, cases of acute abdomen in COVID-19 patients associated with vascular infarctions have increased. Coagulopathy may be present even without clinical respiratory manifestations of the disease. Patients meeting disseminated intravascular coagulation criteria or with markedly elevated D-dimer may benefit from anticoagulant therapy.

**Conclusion:**

Clinicians should suspect of abdominal visceral infarctions in COVID-19 patients presented with acute abdominal pain, despite the absence of respiratory symptoms.

## Introduction

The COVID-19 pandemic is a challenge for health systems worldwide. Although a majority of infected patients remain asymptomatic or have mild to moderate respiratory disease, several complications and new clinical presentations related to SARS-CoV-2 infection are described every day [1,2].

Multiple studies suggest that SARS-CoV-2 infection is associated with a pro-thrombotic state and an increased risk for venous and arterial thromboembolism [3,4]. Among the most commonly found abnormal coagulation parameters are elevated D-dimer and platelet count with low antitrombin levels [5].

Thrombotic events have been recorded in several organs and systems: pulmonary embolism has been the event most commonly associated with COVID-19. However, more recently, abdominal visceral infarctions have been reported including splenic infarction, renal infarction and intestinal ischemia [5].

Splenic infarction is a rare disorder that can present as left-abdominal pain and may be secondary to hypercoagulable states [5]. To date, clinical cases of COVID-19 with splenic infarction are rare in the literature.

In this article we report a case of a previously healthy patient, with clinical manifestations of an acute abdomen and presenting an extensive splenic infarction after infection by SARS-CoV-2.

## Case report

A 67 year-old man was admitted to the emergency department with a moderate, dull, left-sided abdominal pain. The pain had 12 days of evolution, progressive worsening and was associated with nausea and inappetence. He denied fever, respiratory symptoms or changes in bowel or urinary habits. The patient initially sought the Basic Health Unit due to abdominal pain and positive epidemiology. RT-PCR was performed for SARS-Cov-2, which became positive. He was medicated with simple analgesia, but there was a worsening of abdominal pain, leading the patient to seek medical attention again. He had a history of systemic arterial hypertension. His daily medications included enalapril and hydrochlorothiazide.

On physical examination, the patient appeared well, was alert, communicative and afebrile. He was in no acute distress, and his lungs were clear on auscultation. Lower limbs without edema, palpable and symmetrical pulses in all segments and presence of ocher dermatitis in the left lower limb. His abdomen was soft with mild tenderness in the left upper quadrant in superficial and deep palpation, without peritoneal irritation.

On admission, his complete blood count (CBC) and comprehensive metabolic panel (CMP) were unremarkable. However, he had an elevated D-Dimer of 1523 ng / ml.

**Figs. 1 and 2:**
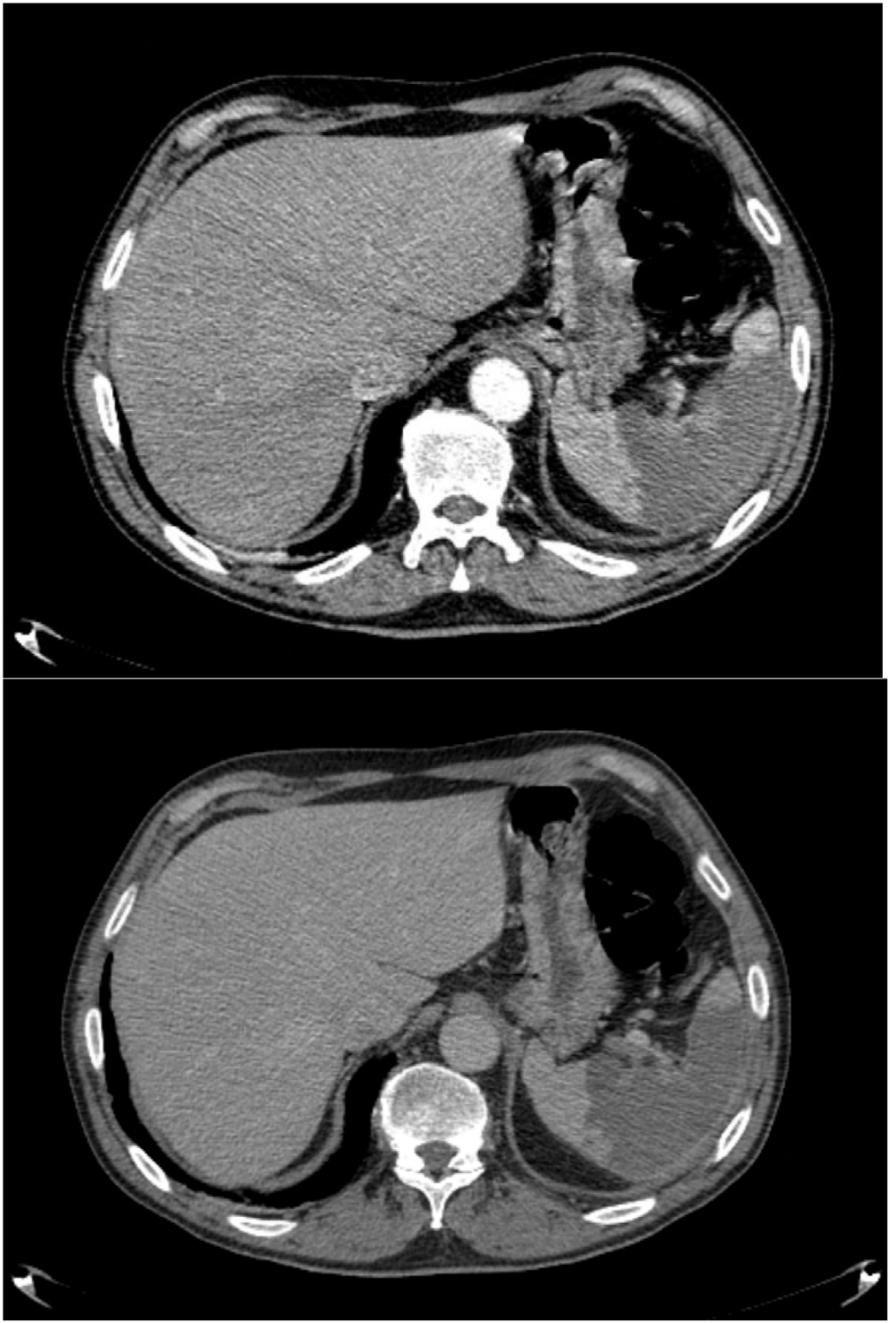
Axial CTA images showing hypodense areas throughout the spleen consistent with splenic infarctions.

**Fig. 3:**
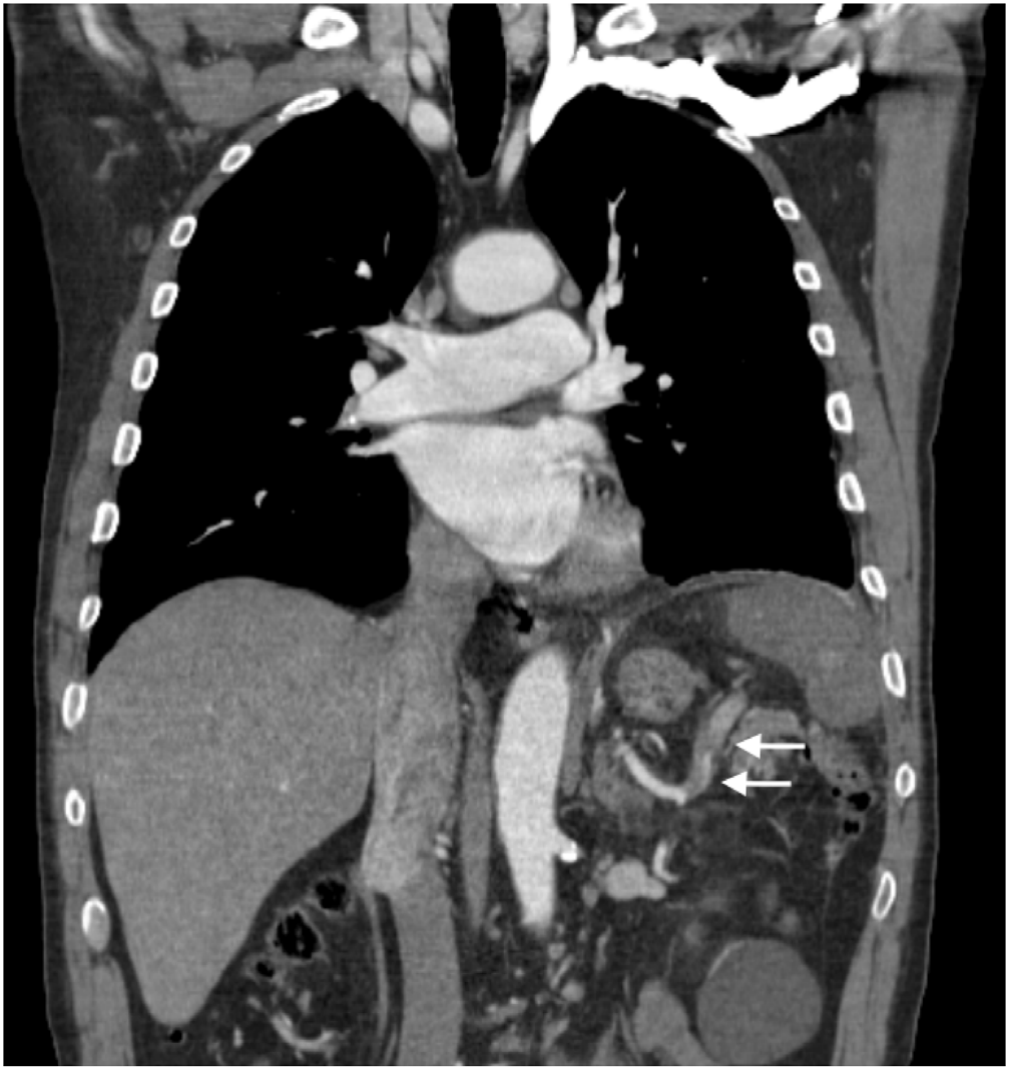
Coronal CTA image showing hypodense areas throughout the spleen consistent with splenic infarctions associated with a thrombus in the distal region of the splenic artery.

**Fig. 4:**
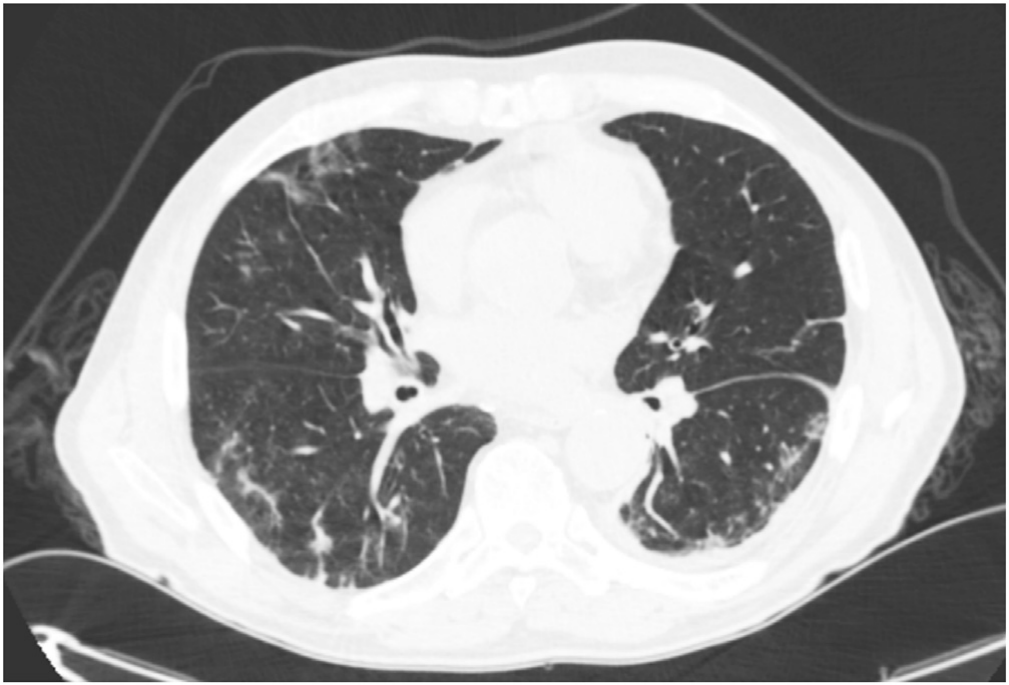
Axial CTA image showing small patchy ground-glass opacities in bilateral pulmonary bases.

A computed tomography (CT) of the chest and total abdomen with IV contrast (Figs. 1, 2, 3) showed the presence of splenic infarction involving about 70 % of the parenchyma with distal thrombus in the splenic artery, absence of lesions of other intra-abdominal viscera and absence of free liquid or pneumoperitoneum. Chest CT (Fig. 4) reveals small, patchy ground-glass opacities in bilateral pulmonary bases, less than 30 % of involvement of the parenchyma, suggestive of SARS-CoV-2 infection.

The patient underwent a routine clinical evaluation for malignancy, followed by a review of the CT images. Chest-abdomen-pelvis CT examination and laboratory markers were all negative for malignancy. The patient also had recent upper endoscopy and colonoscopy, both negative.

He had no clinical signs of gastrointestinal tract infection, nor any other localized infections. Furthermore, he did not present fever, the blood cultures were negative, transthoracic echocardiography was normal, and there was no other clinical sign for endocarditis.

The patient was diagnosed with splenic infarction due to coagulopathy related to SARS-Cov-2 infection. We opted for conservative treatment with full anticoagulation with Enoxaparin1mg/kg twice daily.

The patient’s abdominal pain decreased gradually after anticoagulation and simple analgesia. On day 5, the patient was discharged home on oral Rivaroxaban with outpatient return in 1 month. At the return, the patient was assintomatic. On the follow-up CT the spleen decreased in size and there was no clinical sequelae.

## Discussion

Splenic infarction occurs as a result of occlusion of the splenic artery or its branches due to thrombosis or embolism. The most commonly associated etiologies are cardioembolic events, hematological and neoplastic diseases; but this event has also been associated with acute viral infections, which often trigger states of hypercoagulability [6].

Most patients have abdominal pain in the hypochondrium or left flank. However, as reported by Kranidiotis et al., a surprising percentage of patients may present without abdominal pain [6].

Although thrombotic events are one of the main manifestations related to SARS-CoV-2 infection, reports of splenic infarction are scarce in the literature. Its incidence is probably underestimated, since abdominal imaging tests are not routinely performed and reported cases are often incidental findings from chest CT scans that extend to the upper abdomen [4]. The case described reinforces that the differential diagnosis of abdominal visceral infarctions must be listed within of the spectrum of clinical manifestations of COVID-19-related hypercoagulability.

Klok et al. demonstrated a cumulative incidence of 31 % of thrombotic events in 184 COVID-19 patients admitted to Intensive Care Unit. The use of prophylactic dose low-molecular-weight heparin (LMWH) has been shown to be associates with lower mortality in patients with severe COVID-19 or D-dimer levels more than 6 times the upper normal limit. Societies guidelines support the use of prophylactic doses of anticoagulants in all hospitalized patients with COVID-19, in the absence of a clear contraindication [5]. However, the use of empirical full dose anticoagulation remains controversial in patients without a definitive diagnosis of thromboembolism [7].

Abnormal coagulation parameters have been reported in patients infected with COVID-19. Although the underlying mechanism of COVID-19 coagulopathy remains unknown, it has been suggested that it is a form of disseminated intravascular coagulation (DIC). Recent studies describe that the infection causes the release of a significant amount of pro-inflammatory cytokines, which in some individuals can lead to a systemic inflammatory response syndrome characterized by an increase in serum cytokines that can culminate in prominent hypercoagulability [3]. In addition, evidence accumulated to date suggest that the new coronavirus may be associated with generalized vasculopathy in different systems [1].

Thrombovascular manifestations have been recorded in several organs. Pulmonary thromboembolism is the event most commonly associated with COVID-19. However, recently, several infarctions of abdominal organs have been reported as splenic infarction, renal infarction and intestinal ischemia [5]. Although rare in the literature, cases of acute abdomen in COVID-19 patients associated with vascular infarctions have increased [4,7].

Ramathan et al. describes a case of a patient with concomitant renal and splenic infarction with good clinical evolution after anticoagulant therapy. This study reviews international literature with 13 patients presenting splenic or renal infarctions. Regarding epidemiology, the mean age of patients was 62 (46–72) years, mostly male (92 %) and the most common pre-existing condition was systemic arterial hypertension [7].

In a series of cases with patients infected with SARS-CoV-2 in Brazil, patients had respiratory symptoms associated with splenic, pulmonary and brain infarctions, confirmed by imaging tests [4]. In splenic necropsies performed on 10 patients who died from COVID-19 in Wuhan, China, one case showed arterial thrombosis and splenic infarction [8].

Martin-Rojas et al. relates coagulation disorders with worse prognosis in COVID-19. In their study, D-dimer levels were significantly elevated in patients with an unfavorable outcome (mean 1472.5 vs 385 ng / ml; p = 0.004) [9].

Agha et al. report a patient with splenic infarction despite the use of intermediate-dose LMWH in a morbidly obese patient with a severe case of COVID-19 [5]. Tang et al. suggests that anticoagulants may not benefit the unselected patients, but patients meeting disseminated intravascular coagulation criteria or with markedly elevated D-dimer may benefit from anticoagulant therapy, mainly with LMWH. However, further data from prospective studies is still need to support full-dose anticoagulation in COVID-19 [10].

Splenic infarctions can progress with complications such as abscess or massive hemorrhage causing hypovolemic shock. Karbi et al. report a case of spontaneous hemoperitoneum associated with splenic infarction in a COVID-19 patient. However, these events are rare [2].

## Conclusion

In the present report, we describe a case of splenic infarction in a patient with non-respiratory manifestations of COVID-19, which reinforces that coagulopathy may be present even without clinical respiratory manifestations of the disease.

Clinicians should suspect of abdominal visceral infarctions in COVID-19 patients presented with acute abdominal pain.

## Funding

This research did not receive any specific grant from funding agencies in the public, commercial, or not-for-profit sectors.

## Consent

Written informed consent was obtained from the patient for publication of this case report and accompanying images. A copy of the written cnsent is available for review by the Editor-in-Chief of this journal on request.

## CRediT authorship contribution statement

**Gustavo Rodrigues Alves Castro:** Supervision, Project administration, Writing - review & editing, Resources. **Iwan Augusto Collaço:** Supervision, Project administration. **Caroline L. Balcewicz Dal Bosco:** Visualization, Project administration, Writing - original draft, Writing - review & editing, Conceptualization. **Gustavo Gusso Corrêa:** Conceptualization, Data curation, Resources, Writing - original draft. **Giovana Balcewicz Dal Bosco:** Conceptualization, Writing - original draft. **Giovana Luiza Corrêa:** Conceptualization, Writing - original draft.

## Declaration of Competing Interest

None.
